# A Groundbreaking Insight Into Primary Care Physiotherapists’ Remuneration

**DOI:** 10.7759/cureus.54732

**Published:** 2024-02-22

**Authors:** Athanasios Psarras, Stefanos Karakolias

**Affiliations:** 1 Department of Physiotherapy, Rehabilitation Center Anabiosi, Drama, GRC; 2 Department of Organisation Management, Marketing and Tourism, International Hellenic University, Thessaloniki, GRC

**Keywords:** physiotherapy, cost-per-case, co-payments, preferences, remuneration

## Abstract

Introduction

Physiotherapy in Greece, as part of primary health care (PHC), faces sound imbalances: reduced quality, productivity, and efficiency, along with rather inflexible remuneration schemes. This study is aimed at reporting the attitude and perceptions of Greek PHC physiotherapists toward their current remuneration and also at identifying any other preferable remuneration schemes.

Methods

A stratified proportional sampling study was undertaken, using an anonymous, electronic survey. The participants were 250 self-employed physiotherapists running their business in Central and Eastern Macedonia and Thrace, being also contracted with the National Organisation for Healthcare Provision (EOPYY). The sample size stands for 34% of the population with a circa 5% margin of error.

Results

Nearly 9/10 physiotherapists (84%) underline that remuneration falls short of their productivity, leading to reduced job satisfaction. Moreover, their remuneration does not motivate them to provide services of higher quality (46%), while 58% of them stated that they are forced to claim informal fees. There is no clear desire regarding the remuneration scheme, but nearly ¼ of physiotherapists revealed their preference for the cost-per-case philosophy combined with co-payments.

Conclusion

The majority of physiotherapists believe that their current remuneration does not reflect their productivity nor the quality of their services and, therefore, informal payments arise. The preference of physiotherapists lies between cost-per-case fees and patient co-payments, which, however, favors supplier-induced demand and access inequalities, respectively. Hence, policymakers should revise the current remuneration scheme and overcome its deficiencies without creating new ones.

## Introduction

Physiotherapy (or physical therapy) is a particularly vital sector of health care worldwide. The physical therapy profession in Greece was practiced under the supervision and guidance of doctors, until 2014, when the profession was officially recognized as an autonomous science. Afterward, physiotherapists legally evaluate and choose for themselves the practices they apply to their patients. Physiotherapy is part of primary health care (PHC), which, however, faces many problems in Greece [1]. In addition to accessibility issues, there are also significant remuneration issues [2]. Until 2011, physiotherapists were paid only on a fee-for-service (FFS) basis, with each practice being paid differently. This remuneration model caused significant problems in terms of public expenditure, as the phenomenon of supplier-induced demand was particularly inflated [2-5]. In 2011, with the advent of the financial crisis and then with the establishment of the National Organisation for Healthcare Provision (EOPPY), an effort was made by the local government to limit health costs, with the physiotherapy sector not being left out [6-9]. The remuneration model is changing, and now, self-employed physiotherapists are paid via insurance funds for each patient treatment session, as it happens in other health systems [10]. However, a closed budget has been established for this sector, and, when it is exceeded, it leads to clawback remuneration cuts [11]. In these years, an effort was made to improve the rules of remuneration, by guaranteeing a legal financial participation of the insured patients in all treatments. However, the authorized supplementary private contributions from patients prove insufficient to meet the requisites of physical therapists, and it is still hard for physiotherapists to provide a good and financially viable treatment that a person is willing to pay for [12]. Thus, many physiotherapists are facing sound financial, time, and productivity challenges.

In recent years, important steps have been taken to upgrade PHC, targeting its physicians [13,14]. No public studies have been conducted on the remuneration and job satisfaction that physiotherapists receive. The present study-research aims at collecting and reporting the opinions of Greek physiotherapists employed in PHC, regarding their remuneration, and determining if any internationally applied remuneration model would probably be preferred.

## Materials and methods

The operation of PHC in Greece is particularly problematic, and the remuneration models are quite inflexible [2]. FFS is the prevailing reimbursement for private health care providers, while public providers are salaried [1]. The described financial situation combined with the applied remuneration schemes does not provide significant motives to increase efficiency and productivity [1,2,12]. In studies conducted in the past and more specifically among PHC doctors, it was found that almost all of them express great dissatisfaction with the current remuneration scheme, but also with the amount of their remuneration [1]. In the case of physiotherapists, even though there is evidence of their job satisfaction [15], there are no previous public surveys focused on their remuneration. However, it seems that this specific group of health professionals is not satisfied enough with the level (or even the type) of their remuneration, as the official protests and participation in strikes of the Panhellenic Association of Physiotherapists (PAP) are frequent [14]. Based on the above, the main research hypotheses are first that the existing remuneration model is an obstacle to the provision of quality health services and PHC physiotherapists, as they are dissatisfied with their existing remuneration model, and, second, they would probably prefer a different model.

Study design

A primary stratified proportional sampling survey was conducted using a bespoke anonymous electronic survey.

Participants

The survey sample size was calculated based on the Neyman allocation at 34% of the population. In total, 250 responses were collected from the 734 physiotherapists in the population, with a circa 5% margin of error. The same proportion, of 34%, was approximately maintained in each stratum-regional section. The collection of data took place in the period between 01/09/2022 and 15/11/2022. The inclusion criteria were the following: physiotherapists must have been self-employed, maintained a physical therapy laboratory, been contracted with EOPYY, and operated in the regions of Central Macedonia, Eastern Macedonia, and Thrace. The exclusion criteria were the following: physiotherapists of the public sector and any physiotherapist who is being compensated with salary.

Survey development

A bespoke survey was developed based on a previous survey that was developed for analyzing Greek PHC doctors’ remuneration [1]. The survey included 18 items across three domains: demographics, evaluation of the current remuneration system, and preferences in remuneration systems. Multiple choice closed questions were used, including Likert scales.

Data management and statistical analysis

Data analysis was performed using Statistical Product and Service Solutions (SPSS) (version 25.0; IBM SPSS Statistics for Windows, Armonk, NY). Descriptive statistics analysis, cross-tabulation, and χ2 chi-square tests were performed to interpret the results and draw conclusions. Statistical significance was set at P < 0.05.

## Results

Demographics

About 250 physiotherapists participated in this survey. Among them, 139 (56%) were male, and the remaining 111 (44%) were female, with most of them being between 22 and 30 years old. Detailed demographic data are provided in Table 1.

**Table 1:** Demographic data

	Participants (n=250)	Percentage (%)
Q1. Sex		
Male	139	55.6
Female	111	44.4
Q2. Age		
22-30	72	28.8
31-39	67	26.8
40-48	64	25.6
49-57	31	12.4
58-66	15	6.0
>60	1	0.4
Q3. Marital status		
Married without children	20	8.0
Married with children (up to two children)	106	42.4
Married with children (>= three children)	18	7.2
Single parent family	9	3.6
Single or divorced without children	97	38.8
Q4. Physical therapy specialization		
Sports physical therapy	29	11.6
Cardiovascular and respiratory physical therapy	3	1.2
Neurological physical therapy	19	7.6
Geriatric physical therapy	10	4.0
Physiotherapy in women's health	4	1.6
Pediatric physical therapy	25	10.0
Manual therapy	74	29.6
Physiotherapy of the lymphatic system	8	3.2
Not specialized	78	31.2
Q5. Years qualified		
< 10	109	43.6
11-20	75	30.0
21-30	49	19.6
31-40	16	6.4
> 41	1	0.4

Evaluation of the current remuneration system

As illustrated in Table 2, nearly nine out of 10 physiotherapists (84%) agree that their remuneration does not match the produced work, subsequently leading to low job satisfaction. Most physiotherapists took a neutral stance on whether their remuneration was lower than that of their colleagues of different specializations (46%). Moreover, they believe that their remuneration does not motivate them to provide services of higher quality (46%). Conversely, 54% of them (21% neutral and 33% disagreement), believe that their remuneration does not affect the quality. Therefore, it is evident that, even though a considerable number among them have faced drawbacks in providing superior services, an equally significant proportion (54%, combining neutral and negative aspects) has been able to uphold the offered quality. Remuneration does not motivate physiotherapists to increase their productivity, at 46%, while 31% answered that their productivity is not affected. As previously, it seems that, while there is a large percentage whose productivity has been affected, an equally large percentage of 54% (including neutral responses) has managed to maintain or even increase productivity, with men being the ones who feel that their remuneration affects them negatively to a much greater extent than women (p = 0.018 < 0.05 in Table 3). Moreover, 58% of the respondents stated that they are forced to claim illegal informal fees from patients. Among them, men are forced to claim informal fees from patients to a much greater extent than their female colleagues (p = 0.001 < 0.05 in Table 3), while less-experienced physiotherapists stated that their remuneration does not motivate them to claim informal fees compared to those with more experience (p = 0.016 < 0.05). This seems to be a common practice among physiotherapists in Greece, as they try to replace their "lost" income because of clawback [11].

**Table 2:** Evaluation of the current remuneration system ^a^1: strongly disagree; 2: disagree; 3: neither agree nor disagree; 4: agree; 5: strongly agree ^b^No missing values (n = 250)

	Likert scale levels^a^				
	1	2	3	4	5
My remuneration	% of the total (n)^b^				
Q6. Is lower than the work produced	2 (4)	4 (9)	10 (26)	28 (71)	56 (140)
Q7. Is lower than that of different specialty colleagues	6 (16)	9 (21)	46 (116)	26 (64)	13 (33)
Q8. Does not motivate me to provide higher-quality services	16 (41)	17 (42)	21 (53)	19 (47)	27 (67)
Q9. Does not motivate me to increase productivity	17 (42)	14 (35)	23 (58)	18 (44)	28 (71)
Q10. Motivates me to claim informal fees from patients	17 (43)	7 (17)	18 (44)	23 (58)	35 (88)
Q11. Motivates me to have another (illegal) employment	46 (115)	12 (31)	19 (46)	10 (25)	13 (33)
Q12. Motivates me to acquire specialization or further specialization	11 (29)	12 (31)	28 (69)	21 (52)	28 (69)
Rebate and clawback					
Q13. Have reduced the provided quality of physical therapy services	19 (47)	12 (30)	22 (57)	15 (37)	32 (79)
Q14. Have negatively affected my personal and family planning	7 (17)	4 (11)	19 (47)	15 (37)	55 (138)
Q15. Include satisfactory incentives to increase productivity	1 (3)	2 (5)	18 (46)	21 (54)	58 (144)

**Table 3:** Cross-tabulation and chi-square tests

Cross-tabulation	Asymptotic significance (two-sided)
Ε1 * Ε8	0.018
Ε1 * Ε10	0.001
Ε1 * Ε11	0.027
Ε1 * Ε13	0.018
Ε1 * Ε14	0.006
Ε2 * Ε11	0.049
Ε2 * Ε13	0.046
Ε2 * Ε14	0.007
Ε3 * Ε6	0.006
Ε4 * Ε8	0.012
Ε4 * Ε10	0.002
Ε5 * Ε10	0.016
Ε5 * Ε14	0.001
Ε1 * Ε16	0.001
Ε2 * Ε16	0.001
Ε3 * Ε16	0.001
Ε4 * Ε16	0.011
Ε5 * Ε16	0.001

On the other hand, 58% of the respondents state that their pay does not motivate them to have another job. It was observed that men disagree about working in parallel with other work, which may be illegal, in much higher percentages than women (p = 0.027 < 0.05). Physiotherapists answered that their remuneration motivates them to claim some sort of specialty or further specialize (49%). This outcome may be because the acquisition of specialization also entails claiming higher fees [16]. However, there is also a neutral and negative percentage that is comparatively quite large (51%), which can be justified by the fact that even the acquisition of specialization or further specialization will not be able to bring higher fees, as the remuneration from EOPYY is determined for each patient regardless of the physiotherapists’ performed actions.

Rebate and clawback measures have led to a decrease in the quality of provided physiotherapy services, as indicated by 46.4% of respondents. Notably, nearly seven out of 10 physiotherapists specializing in pediatric physiotherapy assert, in significant proportions, that their remuneration has not adversely impacted the quality of their services (p = 0.012 < 0.05). Furthermore, rebate and clawback measures have been reported to negatively impact personal and family planning by 70%, with male practitioners expressing a significantly higher perception of their influence on planning compared to their female counterparts (p = 0.006 < 0.05). Conversely, physiotherapists in older age groups have conveyed a more pronounced impact on their personal and family planning in comparison to their younger colleagues (p = 0.007 < 0.05). Finally, an overwhelming majority of physiotherapists (78.4%) express the belief that rebate and clawback measures lack sufficient incentives to enhance productivity. It is understood that rebates and clawbacks are clear disincentives for the economic and professional survival of physiotherapists [2]. All answers about the evaluation of the current remuneration system are summarized in Table 2, with cross-tabulation and chi-square test results summarized in Table 3.

Preferences on remuneration systems

**Figure 1:**
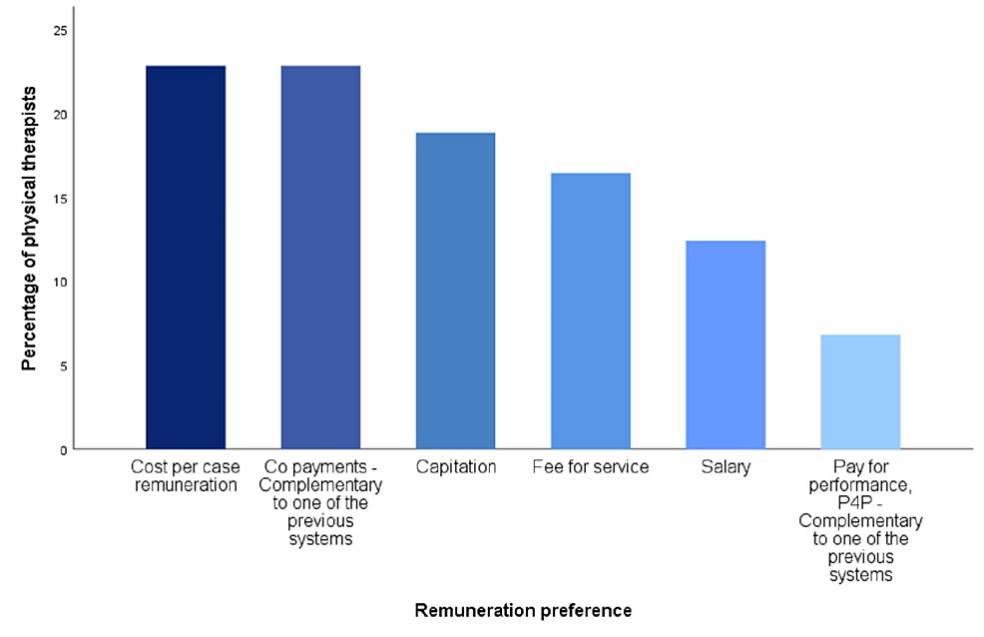
Preferences on remuneration systems

It seems that there is no clear desire regarding the preferable remuneration model among the participants. With the same percentage, 23%, physiotherapists stated that they prefer a remuneration model with co-payments by patients or another remuneration model based on cost, severity, or age, per case. Next comes a capitation model, with a selection rate of 19%, while FFS, which is also the most popular among private sector practitioners, is chosen by 16%. Approximately 12% of the participants chose salary, while only 7% chose the performance-based remuneration model (P4P) [17,18] (Figure 1). Men mainly prefer the co-payment of patients, while women's remuneration was based on cost-per-case (p = 0.001 < 0.05). Female physiotherapists are almost twice as likely as men to prefer cost-per-case as well as salary (31% vs. 19% for cost-per-case and 19% vs. 7% for salary). The two above observations are particularly important, as we observe the different philosophies that the two sexes have, with women tending to safer remuneration methods, while men tending to remuneration methods that have a greater risk (p = 0.001 < 0.05). At ages 22-30, the dominant preferred remuneration models are narrowly cost-per-case and salary (29% and 26%, respectively). Ages 31-39 mainly wish to be paid on an FFS basis (30%). Ages 40-48 and 49-57 want co-payment by patients (38% and 36%, respectively), while the over 58s want capitation model (40%) (p = 0.001 < 0.05). Particularly interesting are the results for the desired remuneration model concerning marital status. More specifically, the highest rates of desire for salary were found among the single or divorced without children. Married people without children find a greater preference for capitation, while those with more than two children equally desire capitation or cost-per-case. Singles primarily want cost-per-case, followed by salary, while married people with up to two children primarily want co-payment (p = 0.001 < 0.05). Physiotherapists who specialize in neurological physical therapy stated that they wish cost-per-case. Those who specialize in pediatric physical therapy choose salary and capitation as the most desirable form of remuneration. In the rest of the specialties, it is observed that the trend of the general population is followed, with those of the general population having slightly increased preferences for capitation (p = 0.011 < 0.05). Cross-tabulation and chi-square test results are summarized in Table 3.

## Discussion

This study highlighted some major challenges of the physical therapy sector in Greece, which are (not surprisingly) aligned with those of the rest of PHC [1]. The financial crisis of 2010 and the memorandums that have been implemented since 2011 have greatly reduced the income of almost all health professionals, including physiotherapists [9,19]. Income falls also had a negative impact on productivity and the quality of their services [2,20,21]. Furthermore, the black economy has been strengthened in all health subsectors with adverse effects on the entire society [11]. However, the public debate about the health sector and PHC is, more often than ever, limited to doctors and nurses. Independent research specifically in the sector of physical therapy does not exist, with the present research trying to open this window, making known to the public the problems physiotherapists face regarding sustainability, fees, and the provided quality. Based on the results, the main research hypothesis seems to be confirmed at first reading. Approximately 46% of the physiotherapists believe that the existing remuneration model (i.e., a fee per session with clawback) is an obstacle to the provision of quality health services, as it does not provide sufficient and direct motivation toward quality. This finding is consistent with that of previous studies on primary care physicians in Greece [1]. Conversely, only 33% of them are against this opinion, while those who are neutral are 21%. However, considering that a neutral attitude means that the quality of the services provided has not been affected to a significant extent, then together with the negative answers in this regard, the percentage rises to 54%, implying that the greater percentage of physiotherapists, whatever problems they face, have managed to maintain the quality of the services provided, unchanged. Hence, in a second reading, we can say that the specific research hypothesis is rejected.

The second main research hypothesis is partially confirmed. The remuneration model with co-payment of the insured is the most dominant (23%); however, with the exact percentage, the model based on cost, severity, and age per case is also preferred. This result shows an inability of physiotherapists to prefer a specific remuneration model and this, most likely, has to do with the complexity of the profession, the different specializations that exist in this sector, the experience of each health professional, and also the personal preferences. This result, of the equality of these two specific models, shows that physiotherapists are anxious and wish to legitimize the informal fees they receive as co-payments from patients so that they can increase them [4]. Simultaneously, the desire for a cost-based payment model shows that there is a desire for physiotherapists to be paid according to the complexity and severity of each case, as in the field of physical therapy, patients come from almost the entire spectrum of medicine, from neurological cases, which most of the time are the most difficult and time-consuming to recover, to the simple cases of muscle injuries or counseling [22]. Another noteworthy discovery is that gender influences payment preferences, as men tend to favor patient co-payment, whereas women show a preference for cost-per-case payment. Age also affects preference, with young people up to 39 years of age wanting a fee-per-case model, while those aged 40 and above show a preference for patient co-payment. Marital status also plays a role, with physiotherapists who are single with no children desiring primarily cost-per-case remuneration but salaried remuneration garnering large percentages as well. Contrarily, married people with up to two children prefer remuneration with co-payment. Specialization could not be missing from the factors that influence the preferences of the remuneration model. Physiotherapists who specialize in neurological physical therapy are the ones who overwhelmingly prefer cost and gravity per case. Those specializing in pediatric physical therapy choose salary and capitation, while, in other specialties, the overall trend is toward patient co-payment and cost-based remuneration.

An additional finding of our study was that the current remuneration model for physiotherapists does not provide sufficient motivation, while it is well-known that FFS (and capitation) rewards intensity and volume of services rather than quality and efficiency [23]. Moreover, we found that the amount of remuneration under the existing model forces physiotherapists to claim informal (illegal) fees from patients, which is deemed a more general deficiency of the Greek health system [24,25]. In the same vein, the respondents were found to attempt to raise their income by acquiring further specialization and, therefore, an international opportunity for relevant reforms such as extending the scope of (physiotherapy) practice emerges [26]. Based on the opinions of the physiotherapists, it is clear that the Greek version of paybacks (i.e., clawback), which was implemented during the financial crisis, had a significant negative impact on productivity, quality, and personal and family planning. This situation seems worse for physiotherapists practicing in urban areas thanks to the very high demand for health services there [27].

The main difficulty we faced during this study was the limited literature regarding the physical therapy sector in Greece. Physiotherapy in Greece has not been studied in depth, and there are no reliable and sufficient public data. Another limitation was that both EOPYY and the official Greek body of physical therapy do not provide up-to-date lists regarding the contracted physical therapists who also maintain a physical therapy laboratory. As a result, reaching 1/3 of the total population was the only feasible choice. Finally, an additional limitation inherent in the study may originate from the potential impact of the economic crisis on the perspectives of young physiotherapists. It is noteworthy that this demographic cohort, being more susceptible to the consequences of the economic downturn, could exhibit altered viewpoints, thereby introducing a potential limitation to the research.

## Conclusions

This specific study was the first independent research in the Greek area focusing on the remuneration scheme applied to physiotherapists. Frustrated with the current remuneration model, physiotherapists want it to change, with patient co-payment and cost-per-case being more prevalent in their preferences. This outcome indicates the endeavor to secure the freedom of physiotherapists to request lawful supplementary fees from patients. Moreover, it reflects their determination and intention to categorize cases based on their severity, recognizing that a physiotherapist may handle numerous cases requiring distinct treatments, which should also be remunerated under an alternative scheme.

Changing the way physiotherapists are paid appears to be a one-way path. The existing remuneration framework does not align with the work produced, prompting physical therapists to claim informal fees from patients. This practice has adverse repercussions on personal and family planning, and there is a notable absence of adequate incentives to stimulate enhanced productivity. A change in the mode of remuneration, with a view of providing motivation to increase productivity and quality of services provided, would help the physical therapy sector get out of the dead end it has entered, significantly increasing healthy competition among physiotherapists. As a suggestion for future research, it is the re-conducting of similar primary research, studying more characteristics of the profession, as well as the comparison of the opinions of physiotherapists according to the annual amount of remuneration they receive. Finally, as the physical therapy sector belongs to PHC, it would be particularly useful to investigate the opinions of physiotherapists regarding the way PHC doctors operate and the degree of agreement with their modes of operation and remuneration.
